# Book Reviews

**Published:** 1891-11

**Authors:** 


### Book Reviews.

A Clinical Text-Book of Medical Diagnosis for Physicians and Students, Based on the Most Recent Methods of Examination. By Oswald Vierordt, M. D., Professor of Medicine at the University of Heidelberg. Formerly Private-docent at the University of Leipzig; later Professor of Medicine and Director of the Medical Polyclinic at the University of Jena. Authorized translation from the second improved and enlarged edition. By Francis H. Stuart, A. M., M. D. With one hundred and seventy-eight illustrations, many of which are in colors. W. B. Saunders, Philadelphia, 1891.

It is with great pleasure that we present a notice of this superior book to our readers. From beginning to end it is filled with the latest and best diagnostic knowledge. Following the introduction come chapters on examination of patients, general examination, examination of respiratory apparatus, examination of the circulatory apparatus, examination of the digestive apparatus, examination of the urinary apparatus, and examination of the nervous system.

The topographical anatomy of each region is concisely presented in the text and clearly delineated in the illustrations. Bacteriology has received due notice by "a concise presentation of those peculiarities of the micro-organisms, whose recognition and discrimination are made possible by cultures and inoculation." "The illustrations of the most important micro-organisms are printed in colors."

Translator and publisher have done their work well. As a whole the book deserves our highest commendation. We heartily recommend it to both student and general practitioner as one of the very best works on medical diagnosis.

F. W. M.

International Clinics. A Quarterly of Clinical Lectures, by Professors in the Medical Colleges of the United States, Great Britain and Canada. Edited by J. M. Keating, M. D., Philadelphia; J. P. C. Griffith, M. D., Philadelphia; J. Mitchell Bruce, M. D., London, and D. W. Finlay, M. D., London. Philadelphia, J. B. Lippincott Co.

The clinical lectures which will appear quarterly in this publication will occupy the entire subject of medicine and surgery. It is the publishers' purpose to make this periodical a complete post-graduate course of medical instruction, and as far as printed lectures can answer for personal observation and contact with patients, we doubt not that these volumes will be an excellent substitute. Among the contributors are the best names in American, Canadian and English medicine and surgery. The subjects are well chosen, and practical, and of great value and interest to the general physician. The lectures on Infantile Eczema, and Modern Methods in Surgical Operations, we may especially commend.

---

Practical Pathology and Morbid Histology. By Heneage Gibbes, M. D., Professor of Pathology in the University of Michigan, etc. Philadelphia, Lea Bros. & Co.

We have not seen a more practical work on microscopical anatomy than this. Parts I and II, on Practical Pathology and Bacteriology, relate to the methods of microscopical examination of tissues and bacteria, and for the purposes designed these subjects are presented in a manner that leaves nothing to be desired.

In Part III, or Descriptive Pathology, the organic lesions of disease are concisely discussed. This portion is fully illustrated, not with diagrams, but with excellent photo-engravings. For those wishing a work of this sort, not able to obtain what is, of course, much better, namely, actual laboratory practice, we know of nothing better.

